# Supplementary material for: Automated stance detection in complex topics and small languages: The challenging case of immigration in polarizing news media
Source: PLoS One. 2024 Apr 26;19(4):e0302380. doi: 10.1371/journal.pone.0302380 (PMC11051607; doi:10.1371/journal.pone.0302380)
Supplement: S1 File — (PDF) [file pone.0302380.s001.pdf]

Immigration related keywords:

Migration (*Ränne*) - captures keywords like “migration” and “migrant”
'migrats|migran|migreer|ränne|rände|rännet|sisserända|sisse rända|väljarända|töörän[dn]|õpirän[nd]|pendelrän[nd]|hiigelrän[nd]|ta gasirän[dn]|massirän[dn]|rändle|rännelnud|tagasipöördu[vj]ad|paadirän[dn]|väljarän[nd]|massipagem|legaal[ns]e[t]*rän[nd]|edasirän[nd]|seaduslik[kult]* rän[nd]|ringirända[vj]|rändajate ümberpaigu|juhitav[a]* rän[nd]|rända[sivad]* sisse|sisse-ja läbiränd|rändajatemass|kodutud ja rändajad|rahvasterän[nd]|rahvaste rän[nd]'
+ additional filter removes mostly animal migration and migraine related sentences:
'lind|linnu|lindu|kala|loom|imetaja|migreen|kahepaik|roomaja|hani|han e|ogavalk|relve|kuula rändajat'

Refugees (*pagulased*) - captures keywords like “refugee”, “asyluym seeker”, “illegal (immigrant)” and “border control”
'pagula|asüül|varjupaigataotl|põgenik|inimkaub|illegaal|piirikontroll '

Foreign workers (*välistööjõud*) - captures keywords like “foreign workers”, “(digital) nomad”
'välistööjõu|tööjõu sisse|võõrtöö|hooajatöö|välismaala|võõramaala|nomaad'

Foreign students (*välistudengid*) - captures keywords like “foreign student”
'Välistuden|välisüliõpila'

Noncitizens (*mittekodanikud*) - captures keywords like “living permit”, “non-Estonian”, “Estonian visa”
'mittekodanikud': 'elamisloa|elamisluba|eesti viisa|viibimisalus|mitte-eestla|muula',

Radical-right vs liberal opposition (*paremäärmus*) - captures keywords like “globalism”, “new-europeans”, “open borders”, xenophobe”, “multicultural”
'globalist|globalism|uuseuroopla|suur asendami|suure asendamise|avatud uste poliitika|avatud piir|ksenofoob|võõrahirm|multikult',

Examples of misclassified sentences

	Human	AI	Probabilities	Example (translated)	Example (original)
**Annotation problems**<br><br>(doubtful annotation)	Pro<br><br>Pro	Against<br><br>Against	[0.7, 0.17, 0.13]<br><br>[0.64, 0.33, 0.04]	I would call on both sides - those who welcome the admission of refugees and those who fear it - not to give their voices to the silent ones.<br><br>President Macron wants to reshape relations between French Muslims and the secular French state.	Kutsuksin mõlemat poolt – nii pagulaste vastuvõtmise tervitajaid kui ka sellega hirmutajaid – mitte andma oma häält vaikijatele.<br><br>President Macron soovib ümber kujundada suhteid Prantsuse moslemite ja ilmaliku Prantsuse riigi vahel.
**Sarcasm**	Against<br><br>Pro	Pro<br><br>Against	[0.11, 0.36, 0.53]	Logically speaking, no ship in the Mediterranean should rescue a migrant ship that is sailing under its own power and not in imminent danger of sinking - bon voyage to Europe!	Loogiliselt võttes ei peaks ükski laev Vahemerel päästma migrandialust, mis liigub omal jõul ega ole otseses uppumisohus – head Euroopasse seilamist!
**Ambiguous and context dependent**<br><br>(Meaning is context dependent, but human reader can infer it from the sentence)	Pro<br><br>Pro	Against<br><br>Against	[0.85, 0.11, 0.04]<br><br>[0.83, 0.11, 0.05]	In Canada, a very pro-multiculturalist and pro-immigration Liberal Party is in power; it's leader and nation's prime minister Justin Trudeau has, for example, promised to raise his sons to be feminists<br><br>The aim of the letter was to make the whole of society unanimously believe that Estonia has no future without Ukrainian migrant workers.	Kanadas on võimul igati multikultuursust ja sisserännet soodustav Liberaalne partei, mille liider ja riigi peaminister Justin Trudeau on lubanud näiteks oma poegadest feministid kasvatada.<br><br>Kirja eesmärk oli panna kogu ühiskond üksmeelselt arvama, et Eesti riigil ilma Ukraina võõrtöölisteta pole tulevikku.

**Third person**<br><br>(sentences talk about someone else's opinion. May often require contextual human knowledge)	Pro<br><br>Pro	Against<br><br>Against	[0.75, 0.15, 0.1]<br><br>[0.58, 0.34, 0.08]	They criticize racism, homophobia, xenophobia and what they see as outdated nationalism.<br><br>In a new Swedish version of "Lyckolandet" ("Happy Land"), Strömstedt expressed anti-racist views and named several people who are anti-immigrant.	Nad kritiseerivad rassismi, homovastasust, võõraviha ja nende arvates vananenud rahvuslust.<br><br>Uute sõnadega rootsi keeles esitatud versioonis „Lyckolandet“ („Õnnemaa“) esitas Strömstedt rassismivastaseid seisukohti ja nimetas mitmeid sisserändevastaseid.
**Other mistakes**<br><br>(sentences for which we could not ascertain causes of mistake)	Against<br><br>Pro	Pro<br><br>Against	[0.05, 0.13, 0.83]<br><br>[0.87, 0.1, 0.04]	As an allied country, Estonia should also offer help in the forthcoming securing of the US southern border against the invasion of illegal immigrants, says Blue Dawn.<br><br>The tragic fate of refugees and their journey to Europe is the number one news story everywhere in the world, yet we have discovered that hate-mongers and provocateurs are disrupting intelligent debate rather than trying to find solutions.	Liitlasriigina peaks Eesti pakkuma abi ka eesseisval USA lõunapiiri kindlustamisel illegaalsete immigrantide sissetungi vastu, leiab Sinine Äratus.<br><br>Pagulaste traagiline saatus ja teekond Euroopasse on kõikjal maailmas uudis number 1, sellest hoolimata oleme avastanud, et vihakõnelejad ja provokaatorid segavad arukat debatti, mitte ei püüa leida lahendusi.

Detailed data distributions

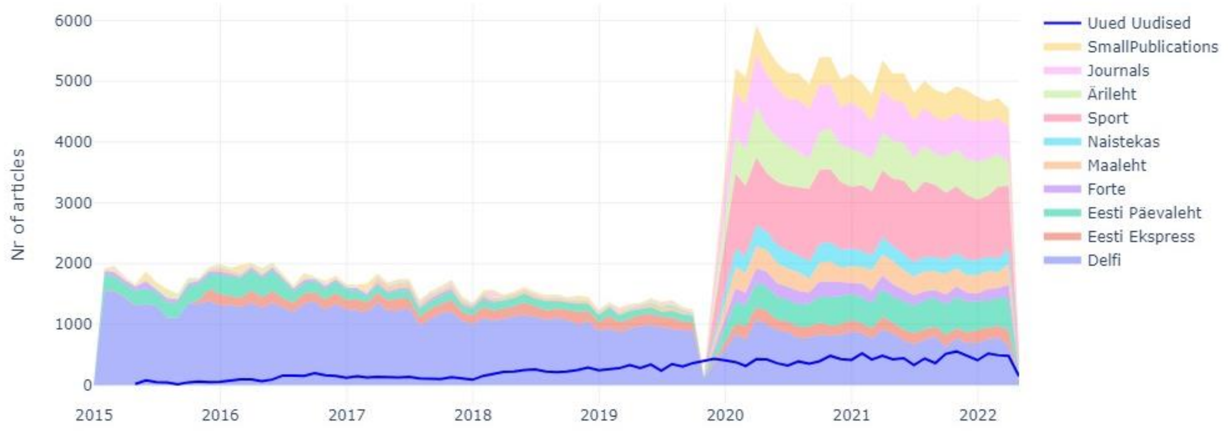

Distribution of all articles in our dataset per periodical. Area chart distinguishes the largest periodicals of Ekspress Grupp per month and the blue trend compares it to Uued Uudised. All Ekspress Grupp articles from 2015 to 2020 mostly originates from Delfi, a fully online platform. From 2020 onwards, the data contains almost all of the other biggest papers as well, including daily newspaper Eesti Päevaleht, weekly news Eesti Ekspress, a Sports portal, Maaleht focusing on rural topics, Ärileht on business and Forte on Technology. The graph excludes numerous other smaller publications that make up a relatively small amount of data. The difference comes from the fact that this data was provided by Ekspress Grupp.

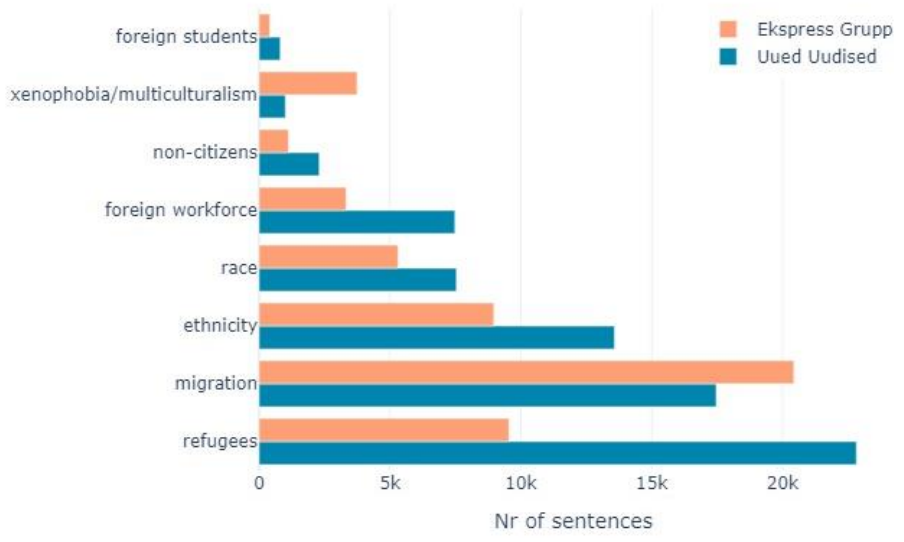

Distribution of keyword groups by the number of sentences mentioning keywords relevant to the group. The refugee and migration related keywords make up most of the dataset whilst there are relatively very few sentences about foreign students. The two outlets have some

differences. E.g. Uued Uudised has double the number of sentences on refugee and foreign workforce topics. Thirdly, xenophobia and multiculturalism related keywords are more used in Ekspress Grupp although there are differences in specific keywords. This indicates that these publishers have different focus on immigration related topics.

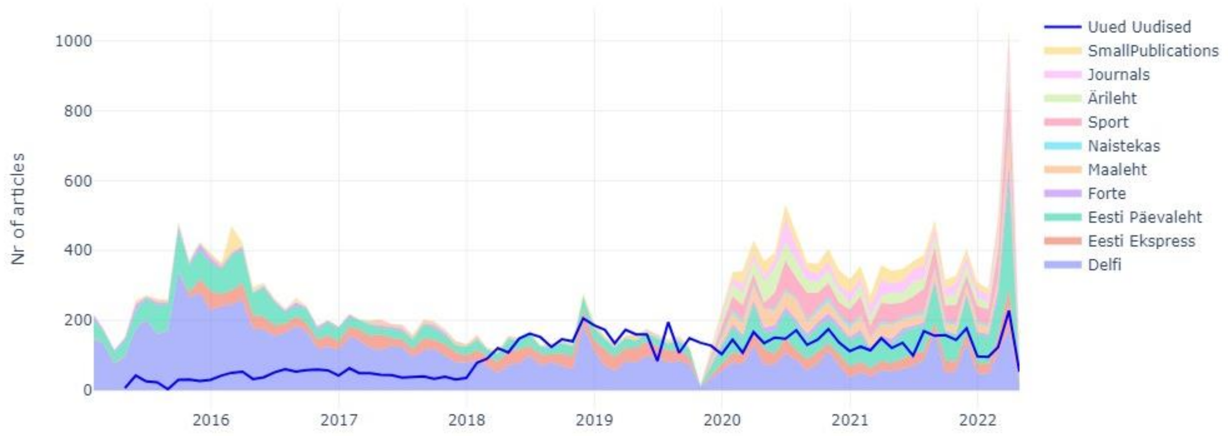

Distribution of immigration related articles per periodical. Shows the articles with immigration related keywords per largest periodicals in Ekspress Grupp in our dataset. Stacked colors represent different publications in Ekspress Grupp. The main source is the online platform Delfi (large purple area). Compared to Uued Uudised marked with blue lines.

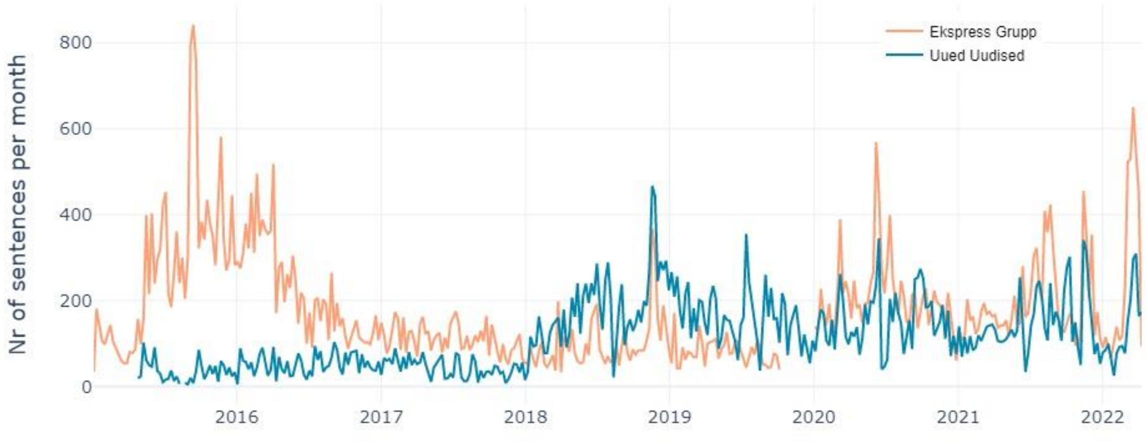

Weekly distribution of immigration related sentences. A more detailed view of immigration related events on a weekly scale. Provided as a comparison to monthly trends which shows shorter term changes behind monthly ones.

Detailed results of models

**Est-RoBERTa**			
	**f1-score**	**precision**	**recall**
**Against**	0.74	0.70	0.79
**Neutral**	0.55	0.50	0.63
**Supportive**	0.69	0.76	0.63
**Micro avg**	0.69	0.69	0.69
**macro avg**	0.66	0.65	0.68
**weighted avg**	0.69	0.70	0.69
**EstBert**			
	**f1-score**	**precision**	**recall**
**Against**	0.69	0.71	0.67
**Neutral**	0.53	0.51	0.56
**Supportive**	0.70	0.70	0.70
**Micro avg**	0.67	0.67	0.67
**macro avg**	0.64	0.64	0.64
**weighted avg**	0.67	0.67	0.67
**XLM-RoBERTa**			
	**f1-score**	**precision**	**recall**
**Against**	0.73	0.70	0.75
**Neutral**	0.54	0.45	0.69
**Supportive**	0.65	0.74	0.58
**Micro avg**	0.66	0.66	0.66
**macro avg**	0.64	0.63	0.67

**weighted avg**	0.66	0.68	0.66
**Multilingual BERT-UNCASED**			
	**f1-score**	**precision**	**recall**
**Against**	0.64	0.61	0.67
**Neutral**	0.38	0.32	0.48
**Supportive**	0.58	0.67	0.52
**Micro avg**	0.57	0.57	0.57
**macro avg**	0.54	0.53	0.56
**weighted avg**	0.57	0.60	0.57
**Multilingual BERT-CASED**			
	**f1-score**	**precision**	**recall**
**Against**	0.66	0.70	0.62
**Neutral**	0.40	0.34	0.48
**Supportive**	0.64	0.66	0.62
**Micro avg**	0.60	0.60	0.60
**macro avg**	0.56	0.57	0.58
**weighted avg**	0.61	0.62	0.60
**ChatGPT**			
	**f1-score**	**precision**	**recall**
**Against**	0.74	0.87	0.64
**Neutral**	0.64	0.56	0.75
**Supportive**	0.57	0.56	0.58
**Micro avg**	0.67		
**macro avg**	0.65	0.66	0.66
**weighted avg**	0.67	0.67	0.69

**Sentiment (Est-RoBERTa)**			
	**f1-score**	**precision**	**recall**
**Against**	0.63	0.49	0.87
**Neutral**	0.42	0.70	0.30
**Supportive**	0.42	0.41	0.42
**Micro avg**	0.52		
**macro avg**	0.49	0.53	0.53
**weighted avg**	0.49	0.58	

Examples of stance annotations

**Stance class**	**Nr of sentences**	**Example** (translated)	**Example** (Original)
Against (1-2)	n=1149	Massimmigration would be disastrous for Europe and it would not solve anything in the world.<br><br>Migrants who are at least somewhat suspicious must be expelled from Estonia in the interests of security.	Massiimmigratsioon oleks Euroopale hukatuslik ja see ei lahendaks maailmas mitte midagi.<br><br>Vähegi kahtlased siin viibivad migrandid tuleb turvalisuse huvides Eestist välja saata.
Neutral (3)	n=1565	Democrats blame the administration for using pandemic against immigration.<br><br>70% of the company's employees are foreigners.	Demokraadid süüdistavad administratsiooni pandeemia kasutamises sisserände vastu.<br><br>70% ettevõtte töötajatest on välismaalased.
Pro (4-5)	n=484	The process to get a residence permit here was not very complicated.<br><br>The Spanish government announced on Tuesday that it would simplify rules for migrants and the unemployed to get jobs in agriculture during the coronavirus pandemic.	Protsess, et saada siin elamisluba, ei olnud väga keeruline.<br><br>Hispaania valitsus teatas teisipäeval, et lihtsustab reegleid migrantidele ja töötutele põllumajanduses töö saamiseks koroonaviiruse pandeemia ajal.
Ambiguous<br><br>(several viewpoints mixed together OR hard to say OR non-related keyword)	n=4117	One can only wonder - when do Libyans quit and follow the flow of things when Europe is just talking about controlling the migrant crisis but itself just pours oil on fire.<br><br>In the past, Kristiina suffered from frequent migraines, which could paralyze a woman and force her into bed for the day, where the only way to cope was to keep a blanket over her head and her ears closed.	Jääb vaid küsida — millal ka liibüalased käega löövad ja toimuval vabavoolus minna lasevad, kui Euroopa vaid räägib rändekriisi ohjeldamisest, ise aga valab õli tulle?<br><br>Varasemalt vaevasid Kristiinat sagedased migreenid, mis võisid naist halvata ja sundida ta terveks päevaks voodisse, kus ainus võimalus hakkama saada oli hoida tekki pea peal ja kõrvasid kinni.

Sentiment annotations used as comparison from Pajupuu et al., 2016

**Class**	**Number of sentences**	**Example** (translated)	**Example** (Original)
Negative (1-2)	n=1927	The leaders of Estonian Air and the public should understand that the strategy does not work or does not work in the way that it is. In a simplified way, we could say that there are two options: to abolish the company or create a new conception	Estonian Airi juhtdel ning avalikkusel tuleks aru saada, et sellisel kujul strateegia ei toimi. Väga lihtsustatult võib väita, et võimalusi on kaks: firma likvideerida või luua täiesti uus kontseptsioon.
Neutral (3)	n=727	Content wise it is the most complicated and delicate issue that could possibly rise in the doctor-patient relationship.	Sisult on tegemist kõige keerukama ja tundlikuma küsimusega, mis üldse võib patsiendi ja arsti suhetes tekkida.
Positive (4-5)	n=882	He added "he is a very interesting person and his style and music are outstanding. I wish him good luck in America"	«Ta on väga huvitav inimene ning tema stiil ja muusika on väljapaistvad. Soovin talle Ameerikas edu,» lisas ta.
Contradictory	n=552	The allies will come after that. Personal resistance does not warrant success – except in the fairytales – but it is still the only way to keep at least some kind of realistic hope for success.	Liitlased tulevad pärast seda. Isiklik vastuhakk ei garanteeri edu – välja arvatud muinasjuttudes –, kuid on siiski ainus viis, kuidas säilitada mingisugune reaalne edulootus.

Details on annotation process

The annotation was preceded by a pilot study on a small sample. Based on the feedback, we improved annotation classes, instructions given to the annotators and further filtered the dataset. They were instructed to classify. The instructions are described in "Annotations section". To assure the quality of annotations, we met with both of the annotators at the middle and end of the annotation procedure. This allowed to discuss the more difficult parts and how to deal with them. The results showed that despite that, the annotators still had somewhat different understanding of especially Neutral (3) and Ambiguous classes.

Inter-rater scores for annotations

Interrater agreement from a sample rated by third annotator (Cohen's kappa). As the two main annotators do not have an overlapping part, both are compared to a sample from the third annotator.

	6 categories (1-5, NA) n=550 (	4 categories (neg,neut,pos,mh) n=550	3 categories (neg,neut+mh,pos) n=550	3 categories (neg,neut,pos) n=222	2 categories (neg,pos) n=82

Both Annotators	0.45	0.49	0.57	**0.68**	0.97
Annotator-J	0.46	0.52	0.59	**0.69**	0.95
Annotator-N	0.45	0.45	0.54	**0.66**	1

Stance per keyword group

In order to better understand the changes, we looked at the stances per keyword group. As the large changes per month complicated the interpretation, we analysed only the yearly change. Average stance per year was relatively stable across topics and their ranking changed little. Most negative keywords for both outlets were keywords often used in relation to radical-right and liberal opposition, like “multiculturalists”, or “globalists” and secondly the topics related to race. The migration-related keywords that were the most prominent in our data, and therefore contributed the most to the overall changes in stances, ranked in the middle for both of the news sources. Findings suggest that from the types of groups in our filtered dataset, the more negative framing is firstly for race, then nationalities and only thirdly migrants more generally. This finding should be approached with caution as it is based on an average over years and with relatively fixed keywords. Furthermore, the stances per keyword topic are often more nuanced than our current approach can distinguish (Koppel & Jakobson, 2023).

In relation to media events, 2018-2019, for Uued Uudised, all of the keyword groups, except the xenophobia and multiculturalism related one, took a more negative stance. For Ekspress, there were more differences per topic. Smaller increase in general anti-immigration stance came mostly from the keywords related to the large topic of migration in general. There were also topical differences relating to the Ukrainian war in 2022. In Uued Uudised the relative number of negative sentences about immigration increased but many other topics got less negative, potentially showing a shift in focus towards the war and refugees. In Ekspress Group, there is a noticeable sharp decrease in Against stances related to radical-right liberal opposition.

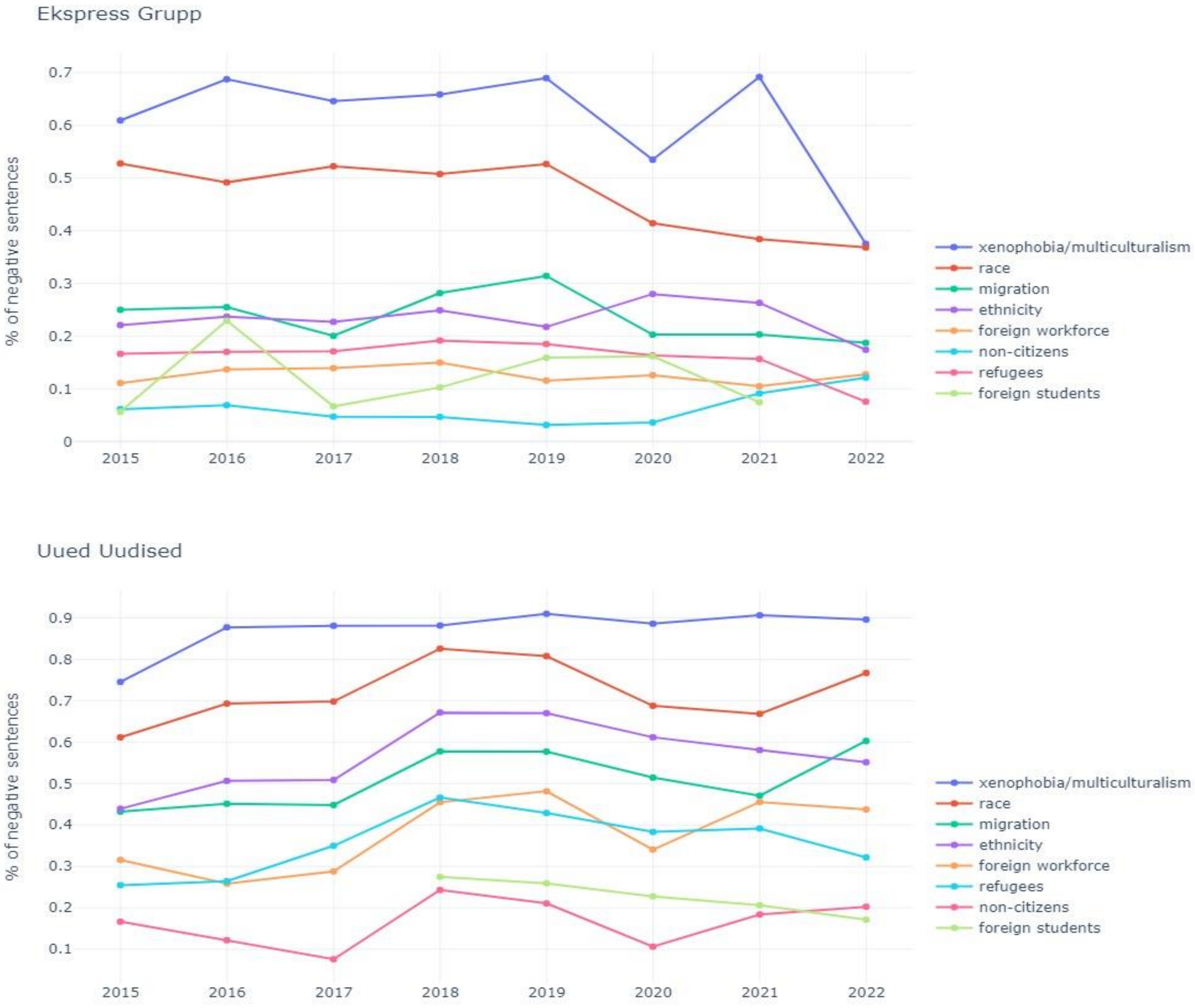

Changes in Against stance per keyword group. Shows the yearly changes per keyword group. We found the Against stance most informative for analysing the changes dependent on specific keyword groups. Notice the different scale of y axis.

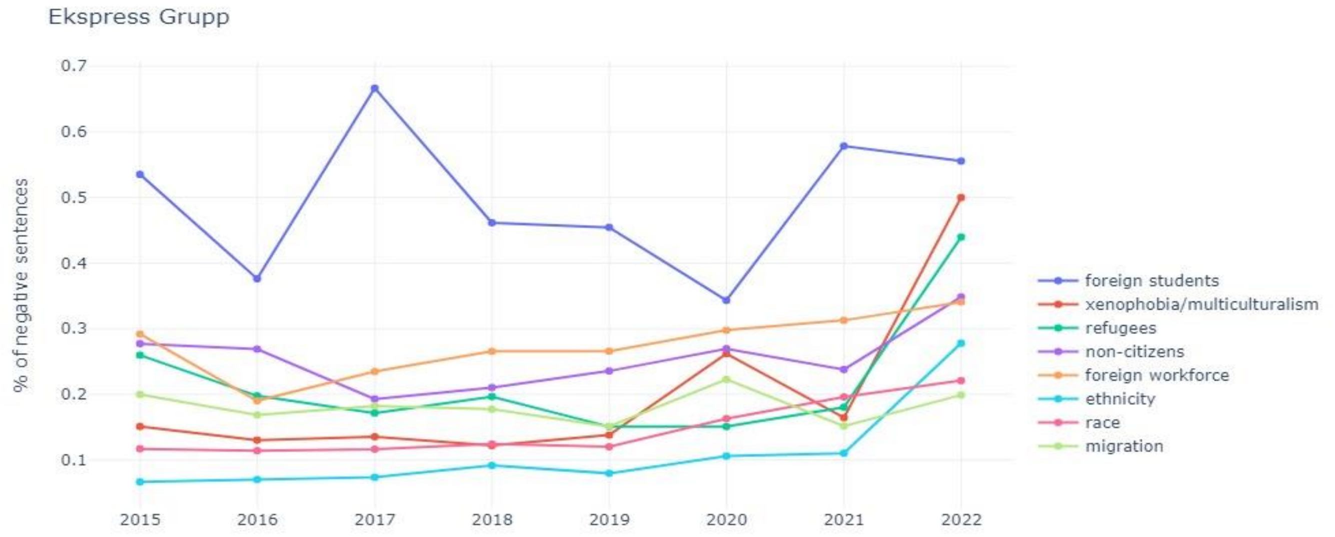

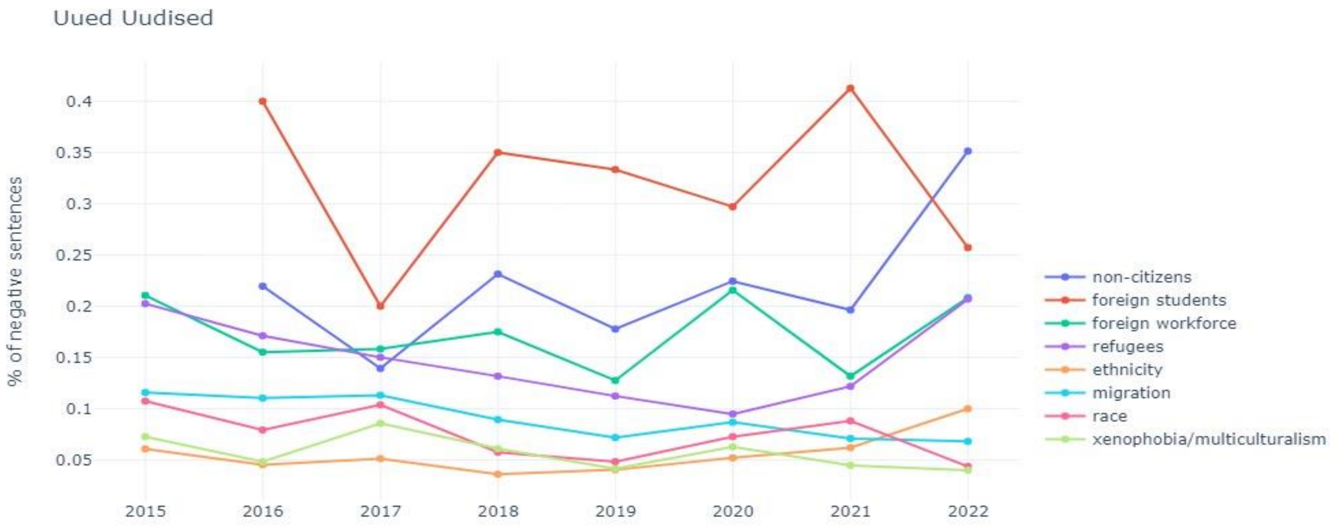

Changes in Against stance per keyword group. Depicts yearly changes. For Ekspress Grupp in 2022 all topics, except foreign students, are getting more positive. Especially xenophobia\multiculturalism related keywords and the large refugee keyword group. Interestingly, there is a slow increase of supportive stances towards foreign workforce across time. There are much smaller changes in 2022 for Uued Uudised for Uued Uudised. But there is more differentiation between topics.

Stance trends with threshold

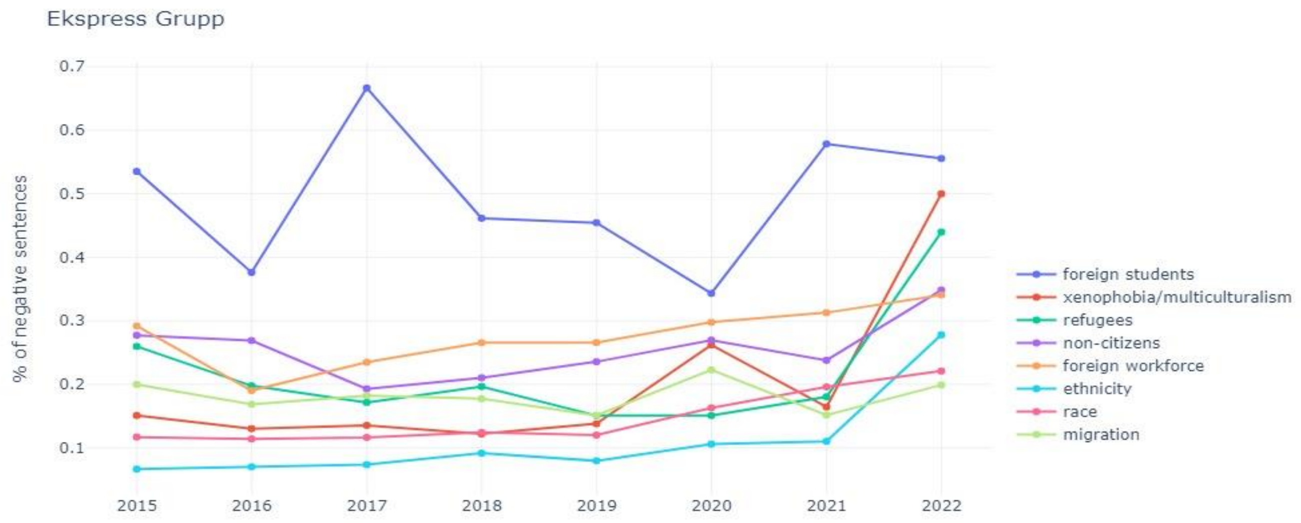

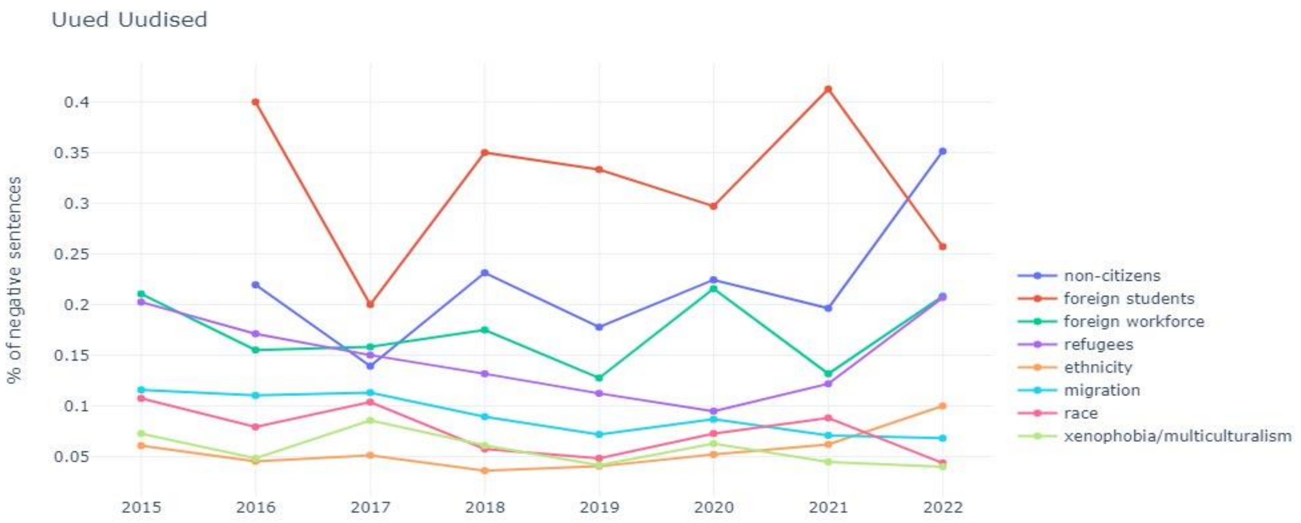

Relative number of stances plus a group of stances that were less uncertain. The uncertain class contains sentences that have less than 70% probability of fitting into a specific class. E.g. a sentence under threshold may have 65% probability of being anti-immigration and the other 35% is shared between neutral and pro-immigration class. The plot shows that using thresholds does not have a large impact on the general trends. Below threshold sentences is relatively larger in Ekspress Grupp than in Uued Uudised, but overall difference is from 5-10% and this variability is relatively small across months.

Sentence embeddings trends

In order to understand the changes taking place within and between the publishers, we calculated the cosine similarities between the sentences from different publishers with Sentence-BERT (Reimers & Gurevych, 2020). Figure 6 shows how the cosine similarity has spiked in the end of 2018 and 2022. We interpret the change as a possible increase of similarities of topics or rhetoric towards immigration. The latter change with the Ukrainian war differs from one connected to the 2018 UN migration pact as the similarity increased almost just as much with all of the stances. Analysis of similarities within publisher sources (figures on next page) had similar trends relating to those events, meaning that both publishers were possibly more focused on one media or used similar rhetoric during these months.

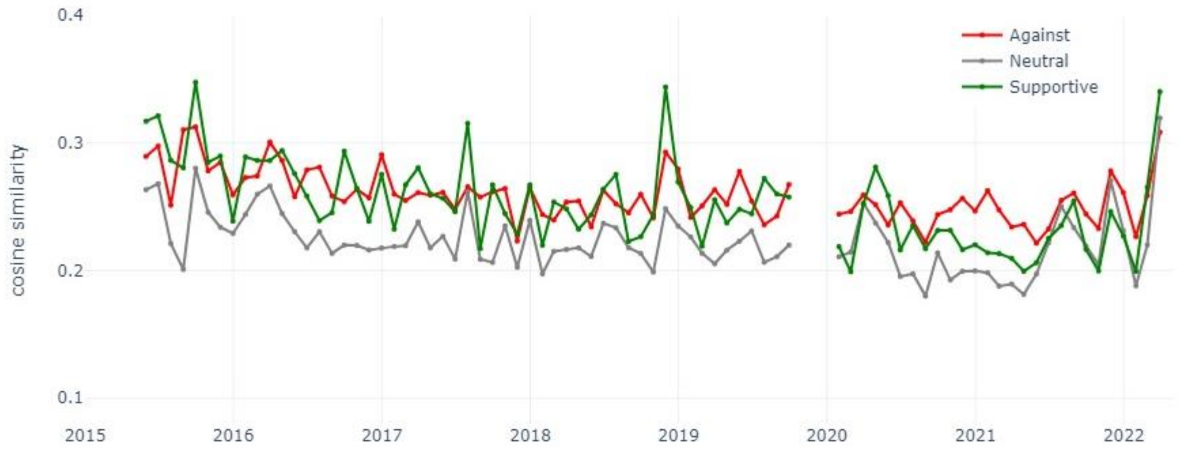

Comparison on sentence cosine similarities between the publishers. Higher value indicates increased similarity between Publishers within that stance group. Similarities are calculated separately per stance. The larger spikes in higher cosine similarities in the end of 2018 and in 2022 may be indicating that the outlets pay attention to similar events in a similar stance.

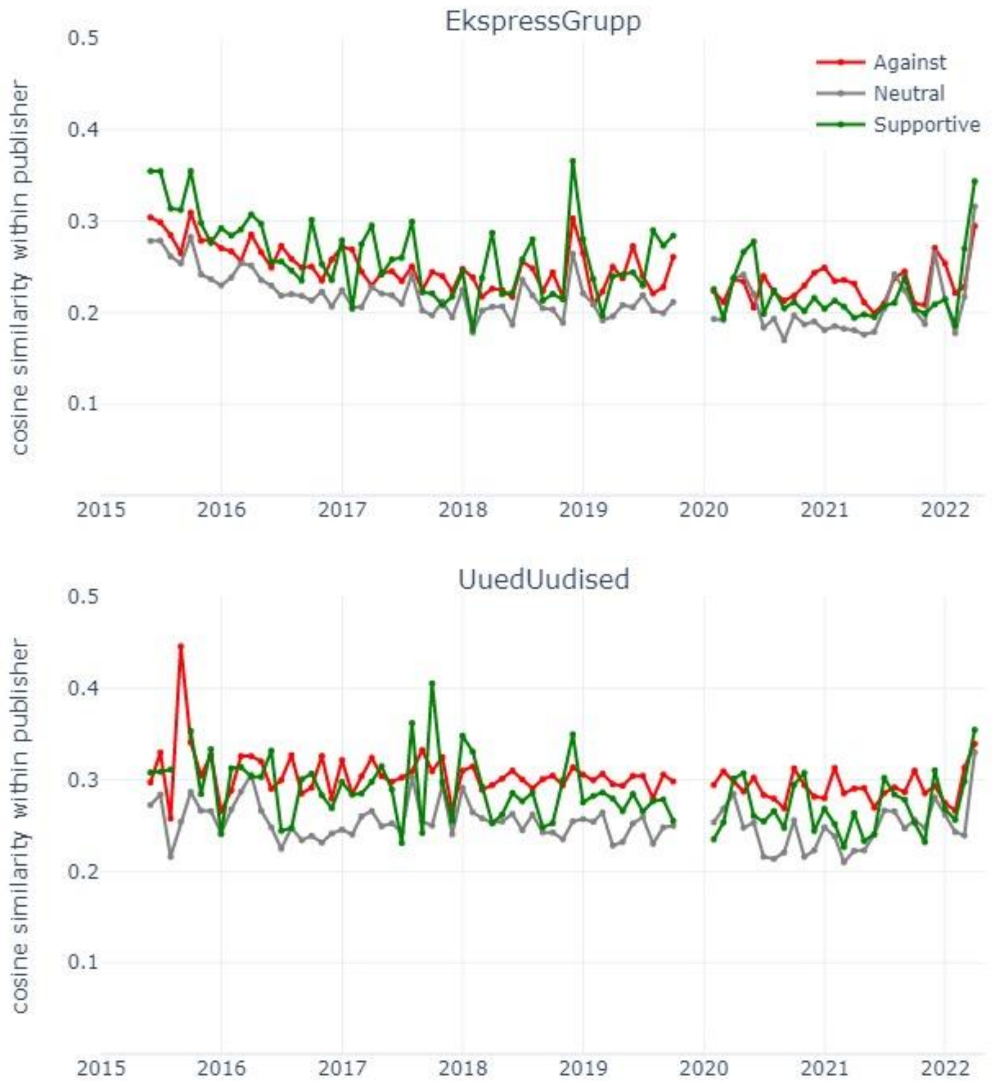

Monthly average cosine similarities of sentences within the same stance. Shows the variation of sentences within stances. Similarities are calculated separately per stance, by comparing all e.g. neutral sentences to all other neutral sentences. The variation in positive stances is expected due to less data.

References

1. Koppel, K., & Jakobson, M.-L. (2023). Who Is the Worst Migrant? Migrant Hierarchies in Populist Radical-Right Rhetoric in Estonia. In M.-L. Jakobson, R. King, L. Moroşanu, & R. Vetik (Eds.), Anxieties of Migration and Integration in Turbulent Times (pp. 225–241). Springer International Publishing. https://doi.org/10.1007/978-3-031-23996-0_13
2. Reimers, N., & Gurevych, I. (2020). Making Monolingual Sentence Embeddings Multilingual using Knowledge Distillation. *Proceedings of the 2020 Conference on Empirical Methods in Natural Language Processing*, 4512–4525.
