# Supplementary material for: Automated stance detection in complex topics and small languages: The challenging case of immigration in polarizing news media
Source: PLoS One. 2024 Apr 26;19(4):e0302380. doi: 10.1371/journal.pone.0302380 (PMC11051607; doi:10.1371/journal.pone.0302380)
Supplement: Attachment — Submitted filename: Response to Reviewers.docx [file pone.0302380.s002.docx]

*Dear Editor,*

*Thank you for the chance to improve our manuscript “Automated stance detection in complex topics and small languages: the challenging case of immigration in polarizing news media”. We found both reviews very useful, and while they included constructive criticism, which we used to improve the paper, we interpreted the reviews as largely positive. Reviewer 1 deemed our topic "important and timely" and said our "research objective is clear", and Reviewer 2 had suggestions for only minor changes. We worked carefully through all of the review suggestions, and followed the suggestions to make the details about the research more explicit. Both reviewers’ comments have been applied in the new version by improving parts of the text, supplementary information and graphs. In addition, we adjusted the formatting to fit PLOS ONE standards. Below we provide responses to all review comments. The reviews are in bold and our answer in normal text under each comment. Thank you for your editorial contribution to our paper!*

*On behalf of the authors,*

*Mark Mets*

**Responses to editorial comments:**

# 1. Please ensure that your manuscript meets PLOS ONE's style requirements, including those for file naming.

We thank you very much for this reminder. We have now changed the manuscript style according to the requirements: 1. The title page has been reorganized with added cities of the authors; 2. Title added to Abstract section and main section titles now have uniform p18 style; 3. Keywords have been removed; 4. Supplementary information has been moved to follow the references and includes a legend; 5. Uploaded supplementary file name will be corrected.

# 2. Please provide an amended statement that declares *all* the funding or sources of support (whether external or internal to your organization) received during this study, as detailed online in our guide for authors at http://journals.plos.org/plosone/s/submit-now. Please also include the statement “There was no additional external funding received for this study.” in your updated Funding Statement. Please include your amended Funding Statement within your cover letter. We will change the online submission form on your behalf.

We updated the Funding Statement and stated that “There was no additional external funding received for this study.”

# 3. We note that the grant information you provided in the ‘Funding Information’ and ‘Financial Disclosure’ sections do not match. When you resubmit, please ensure that you provide the correct grant numbers for the awards you received for your study in the ‘Funding Information’ section.

We confirmed that the funding statement and funding information are matching. The Horizon 2020 grant through the “CUDAN” project is mentioned in both places with the same project nr 810961. The funding from Ekspress Grupp is also mentioned in both places, but there is no grant number to report.

# 4. We notice that your supplementary figures are uploaded with the file type 'Figure'. Please amend the file type to 'Supporting Information'. Please ensure that each Supporting Information file has a legend listed in the manuscript after the references list.

The files will be correctly uploaded as Supporting information. Each supporting information file now has a corresponding legend in the manuscript.

# 5. We notice that your supplementary tables are included in the manuscript file. Please remove them and upload them with the file type 'Supporting Information'. Please ensure that each Supporting Information file has a legend listed in the manuscript after the references list.

The supplementary section has been reorganized according to the journal standards, placed after the references and named as “Supporting Information”.

**Responses to REVIEWER 1 comments:**

**The manuscript explores the application of large language models (LLMs) for automated stance detection in the context of immigration in polarizing news media, focusing on the Estonian language. The study involves annotating a dataset of pro and anti-immigration examples, comparing the performance of multiple LLMs as supervised learners, and investigating diachronic trends over a seven-year period in two corpora of Estonian mainstream and right-wing populist news sources. The article covers an important and timely topic, and the research objective is clear. The use of LLMs for automated stance detection in a challenging scenario is relevant.**

We thank the reviewer for the thorough reading and are happy they agree on the importance and timeliness of the topic!

**However, there are areas where the manuscript could be improved:
1.**	**Abstract: The abstract should provide a concise summary of the study, including the research objective, methodology, main findings, and implications. It currently lacks specific details about the dataset size, performance metrics, and significant findings.**

We agree that an abstract should give a concise summary of the paper, but in this case, as we aim for a more general audience, we would prefer to reserve the more technical details for the relevant sections in the paper. We did improve the abstract to state the results and findings more clearly. We appreciate that there may be differing preferences about abstract specificity in different disciplines.

# 2. Introduction: The introduction provides a good overview of the research context, but more explicit statements about the research gap and the significance of the study must be included. Please clearly articulate how the study contributes to existing literature and highlight the novelty and potential impact of using LLMs for stance detection in lower-resource languages.

We agree and added a new paragraph to the Introduction part that states explicitly which gaps our research fills.

# 3. Methodology:

# a. Provide more details about the annotation procedure, including the guidelines given to the human annotators.

We changed the Annotations section to describe the guidelines for each class.

# b. Explain how inter-annotator agreement was measured and addressed.

The annotations section was improved to better explain the inter-annotator comparison. Additional information is available in Supplementary information.

# c. Also, authors should describe the fine-tuning process of the LLMs, including the hyperparameters used.

We added a more detailed description of the fine-tuning process to the Automatic classifiers subsection. The information about hyperparameters was moved from supplementary information to the main text and placed in the Automatic classifiers section.

# d. Please specify the evaluation metrics employed and discuss their appropriateness for the task.”

We agree this should have been clarified. We added a sentence to the Automatic classifiers section that clarifies the F1 metric we used. F1 is a widely adopted accuracy metric in computer science and adjacent disciplines, so we assume a longer discussion on its usage is unnecessary.

# 4. Results: The authors evaluate the performance of different models and analyze the changes in stances over time. But below are some suggestions for improvement:

# a. Provide more details about the dataset used for training and evaluation. Include information such as the size of the dataset, data sources, and any preprocessing steps applied.”

We agree that this is all relevant information to include. It is indeed already present in the paper in the Dataset subsection, where we describe dataset size, data sources and the preprocessing steps. There are also further details about data distributions and preprocessing in the Supplementary information.

# b. When discussing the performance of different models, you can consider including statistical significance tests to determine if the observed differences are statistically significant.”

We thank the reviewer for the suggestion. As F1 is the most typical approach for the comparison of such computational models, we did not seek to conduct any further statistical tests.

# c. Perhaps you can explain the practical implications of achieving an F1 macro score of 0.66. How does it compare to existing methods or benchmarks in the field? Are there specific applications where this level of accuracy is considered acceptable?

Benchmarking is indeed relevant to validate our findings. However, there are no comparable benchmarks in our example language. We did provide a comparative test with the closest available existing dataset for the similar but not the same task of sentiment analysis. We also discuss work on other languages and domains where similar results have been achieved. In essence, our work constitutes the benchmarking the reviewer is interested in, as we compare a number of different approaches and model architectures.

The practical implications of our model is demonstrated by its application in an exemplary analysis which demonstrates that the inferred trends roughly correspond to known major political changes in this time period. While we are aware of the pitfalls of post-hoc assessment, we see those results as promising and the approach as a potentially useful and applicable tool in large scale media trend analysis. We also point out several other papers that achieve similar levels of accuracy, but also that this is highly task dependent. It is also stressed already in the abstract that we chose to tackle a rather difficult case. Accuracy can be expected to be better in many other cases such as less complex topics and higher-resource languages with larger LLMs.

# 5. Limitations:

# a. Please clarify the limitations attributed to human annotations. What specific challenges were encountered with human annotators? How can these limitations be addressed or minimized in future studies?

We thank the reviewer for turning attention towards a relevant part of the work. We have discussed all of these points of limitation, challenges with specific examples, and future suggestions in the Limitations section.

# b. Discuss the potential biases in the dataset and how they might impact the classifier's performance. You may consider exploring methods to mitigate biases or evaluate the impact of biases on the results.

This is a relevant source of possible error. This is why we pointed out in the Limitations subsection ways to mitigate biases; a major one being related to annotations.

# c. Please provide more details on the annotation instructions given to the human annotators. How were they trained? Did they receive any feedback or clarification during the annotation process? Clear instructions can help improve the quality of annotations.

We added some additional information to the Supporting Information describing the training of annotators and feedback they received. Clear instructions are indeed relevant as also already discussed in the main text.

# 6. Exemplary Analysis:

# 1. I think it is necessary to give a brief overview of the diachronic analysis methodology used for analyzing changes in stances over time. We need to understand the approach taken and the significance of the findings.”

It is a relevant point, but it falls outside the scope of our paper. There are some references provided to similar studies of stance detection with related topics of study. The diachronic analysis of stance detection is already included where these points are discussed. Further elaboration on it might redirect the attention from the paper's main goals. As a solution, we do refer to some papers related to stance detection that can provide a more general overview.

# 2. When discussing the exemplary analysis, more specific interpretations of the findings are needed. For example, what are the implications of the observed trends in stance changes? How do these trends align with existing literature or theories on media polarization and immigration discourse?

We thank the reviewer for the suggestion, this was indeed lacking from the text. We added a paragraph at the end of the section that does exactly that, offers more clear and specific interpretations, implication and relates to some of the existing literature.

# 7. Future Research:

# 1. Elaborate on the suggestion of using generative models like ChatGPT for annotating data or augmenting existing annotations. How would this approach work in practice? What are the potential benefits and challenges?”

# It is an interesting topic to further elaborate. We already provided some discussion, but as this is not the main focus of our paper, we have indeed limited our discussion on that matter. There are forthcoming studies that deal with that in more detail.

# 2. Discuss the potential limitations or risks of relying solely on generative models for annotation. Are there any concerns related to biases or the reliability of annotations produced by these models?

# Similarly to the last comment, this is not the central focus of our paper. However, we do emphasize the importance of expert evaluation, which can be hoped to detect any such possible biases.

# 3. Provide more details on the proposed evaluation of zero-shot learning and the necessity of annotated datasets. How would the accuracy of zero-shot learning be evaluated? In what scenarios would annotated datasets still be necessary?

We have rephrased a part in the Future research subsection that stresses the necessity of annotated datasets for evaluation of zero-shot datasets. It is now clearer that this evaluation should be done like we have demonstrated in our paper.

**Responses to REVIEWER 2 comments:**

# 1. Is the manuscript technically sound, and does the data support the conclusions?

# - “Partly”

Our edits according to the rest of the comments have greatly improved the technical soundness of the manuscript. The statistical significance tests are added, the analysis process itself, possible limitations and conclusions are better worded.

# 2. Has the statistical analysis been performed appropriately and rigorously?

# - “NO”

As mentioned in Reviewer 1 comment 4b, additional significance tests couldbe possible. However, F1 can be regarded as the standard way of comparison. Furthermore, we clarified our process of fine-tuning and evaluation to better show the validity of the statistical analysis. We report cross-validated metrics that assure that the results are not affected by over-fitting during hyperparameter tuning. Yet the weakest part is the annotations, but we have brought out some ways to improve that and now better described the annotation procedure itself.

# 3. Is the manuscript presented in an intelligible fashion and written in standard English?

# - “NO”

We have further proofread the text and improved language and spelling.

# 4. In the introduction section, mention the key contributions of this text/speech classification point by point.

We have improved the introduction section and the key contributions are now more brought out, as mentioned under Reviewer 1 comment 2. We added this paragraph specifically to show our key contributions “We therefore aim to fill multiple gaps in existing research: applications of LLMs both in supervised and zero-shot learning contexts to lower-resource languages, better understanding of far-right populism, media polarization and immigration, and work towards automated trend analysis of these topics, in particular but not limited to the Estonian context.”

# 5. There are a number of classifiers, why did the author choose this particular classifier for this classification problem?

This is brought out in the subsection Analytic approach. We chose it due to its reported effectiveness.

# 6. Classification accuracy is not too high, how this model can be effective in practice?

This is similar to Reviewer 1's comment 4c. While the absolute values of classification accuracy may seem moderate at first, one should consider the complexities involved in the task. If anything, we see this as a very promising result to prompt further research and improvement: The application of our model in a real world case study demonstrates its applicability by matching the trends with major political events and confirming the general expectations. This is also industry-facing research, and our private sector media industry partner considered these results quite promising as well.

# 7. There are some typos and grammatical anomalies in various sections in this article, overall language quality shall be improved.

The text has been proofread and improved as mentioned in Reviewer 2 comment no. 3.

We thank you again for your work with our manuscript!

Best regards,

Mark Mets
